# Decreased mitochondrial‐related gene expression in adipose tissue after acute sprint exercise in humans: A pilot study

**DOI:** 10.14814/phy2.70088

**Published:** 2024-10-21

**Authors:** Mona Esbjörnsson, Håkan C. Rundqvist, Barbara Norman, Ted Österlund, Eric Rullman, Jens Bülow, Eva Jansson

**Affiliations:** ^1^ Division of Clinical Physiology, Department of Laboratory Medicine Karolinska Institutet Stockholm Sweden; ^2^ Unit of Clinical Physiology Karolinska University Hospital Stockholm Sweden; ^3^ Department of Clinical Physiology and Nuclear Medicine Bispebjerg University Hospital Copenhagen Denmark

**Keywords:** biopsy, high intensity, microarray analysis, skeletal muscle, sprint interval exercise, subcutaneous white adipose tissue, transcriptome

## Abstract

The aim was to examine the acute effects of sprint exercise (SIT) on global gene expression in subcutaneous adipose tissue (AT) in healthy subjects, to enhance understanding of how SIT influences body weight regulation. The hypothesis was that SIT upregulates genes involved in mitochondrial function and fat metabolism. A total of 15 subjects performed three 30‐s all‐out sprints (SIT). Samples were collected from AT, skeletal muscle (SM) and blood (brachial artery and a subcutaneous AT vein) up to 15 min after the last sprint. Results showed that markers of oxidative stress, such as the purines hypoxanthine, xanthine and uric acid, increased markedly by SIT in both the artery and the AT vein. Purines also increased in AT and SM tissue. Differential gene expression analysis indicated a decrease in signaling for mitochondrial‐related pathways, including oxidative phosphorylation, electron transport, ATP synthesis, and heat production by uncoupling proteins, as well as mitochondrial fatty acid beta oxidation. This downregulation of genes related to oxidative metabolism suggests an early‐stage inhibition of the mitochondria, potentially as a protective mechanism against SIT‐induced oxidative stress.

## 1 INTRODUCTION

Low‐volume high‐intensity interval training (sprint interval training or SIT) has been shown to reduce body fat mass and waist circumference in (Poon et al., 2024; Viana et al., 2019), in spite of a low energy expenditure during SIT (Brownstein et al., 2022; Macinnis & Gibala, 2017). Such discrepancy between reduction of fat mass and energy expenditure during SIT might be explained by excess postexercise oxygen consumption (EPOC) (Hazell et al., 2012; Panissa et al., 2021), which has been shown to be exponentially related to exercise intensity (Borsheim & Bahr, 2003). Furthermore, a systematic review found that EPOC is higher after SIT than after high‐intensity interval training (HIIT) or endurance exercise (Panissa et al., 2021). Therefore, SIT‐induced EPOC may explain the discrepancy described above.

The question is which physiological stimuli cause the SIT‐induced EPOC. One of the proposed candidates is an increased rate of the triglyceride‐free fatty acid (TG‐FFA) cycle, that is, increased lipolysis followed by increased re‐esterification (Borsheim & Bahr, 2003; Moniz et al., 2020). This cycle is an energy requiring process, in which the energy from consumed ATP disappears as heat, that is, thermogenesis (Brownstein et al., 2022; Townsend et al., 2017; Wolfe et al., 1990). In particular, the energy cost of the TG‐FFA cycle has been estimated to be markedly higher postexercise than at rest (Wolfe et al., 1990). In addition, hormones such as catecholamines and growth hormone stimulate lipolysis (Enevoldsen et al., 2007; Townsend et al., 2017; Yoshioka et al., 2001) and thereby also the TG‐FFA cycle and energy expenditure (Rognstad & Katz, 1966). Based on in vitro experiments, the TG‐FFA cycle is thought to be the major consumer of ATP in adipose tissue in response to catecholamines and growth hormone (Rognstad & Katz, 1966). High‐intensity exercise may enhance this process during the postexercise recovery (Pritzlaff et al., 2000; Yoshioka et al., 2001). Acute SIT is known to induce a profound systemic response, such as an increase in catecholamines and growth hormone in blood (Esbjörnsson et al., 2009; Esbjörnsson‐Liljedahl et al., 2002). Recently, SIT has also been found to increase the release of the cytokine interleukin‐6 from adipose tissue, in parallel with an increased release of glycerol from adipose tissue, indicating an increased lipolysis (Esbjörnsson et al., 2023). Thus, several of the SIT‐induced changes in hormonal and metabolic systems are potential candidates for an increase in lipolysis and thermogenesis in white adipose tissue during postexercise recovery (Enevoldsen et al., 2007; Lange, 2004; Laurens et al., 2020; Rognstad & Katz, 1966; Thompson et al., 2012; Yoshioka et al., 2001). Finally, increased TG‐FFA cycling may in turn stimulate mitochondrial biogenesis by activating AMP‐activated protein kinase (Brownstein et al., 2022; Ceddia, 2013; Gauthier et al., 2008; Townsend et al., 2017). Therefore, the focus of the present study was to elucidate the effects of SIT on gene expression related to mitochondrial and fat metabolism in adipose tissue.

To the best of our knowledge, there are no previous publications on acute effects of SIT on adipose tissue gene expression or metabolic signaling. However, there are a few other studies on the acute effects of exercise at lower intensities than during SIT, but still classified as high intensity and performed either as intervals (Islam et al., 2020; Ludzki et al., 2021) or continuously (Dreher et al., 2023; Fabre et al., 2018). Yet, the results of these studies on gene expression or signaling in adipose tissue are inconclusive. Differences in experimental protocols such as intensity and type of exercise, different populations such as obese versus nonobese individuals and the time between the end of exercise and biopsy may have influenced the results (Guerrier et al., 2023; Ludzki et al., 2021). Only one study was found on the non‐acute effects of SIT on adipose tissue, but the focus of the study was on insulin resistance and vascularization and not on the thermogenic response of adipose tissue to exercise (Honkala et al., 2020).

Therefore, the main aim of the present study was to examine the acute effects of sprint exercise on global gene expression in subcutaneous adipose tissue of healthy subjects to better understand how sprint exercise may contribute to body weight regulation in humans. The hypothesis was that the expression of genes specifically related to mitochondrial function and fat metabolism increases after acute sprint exercise.

## 2 METHODS

### 2.1 Subjects

A total of 15 healthy volunteers (eight females and seven males) participated in the study. Mean ± SD age, height, body weight, and body mass index were 27 ± 7 years, 171 ± 9 cm, 70 ± 11 kg, and 24 ± 2 kg x m^−2^, respectively. Subjects were recruited from a college of sports and recreation instructors. Inclusion criteria were healthy, nonsmoking young adults of both sexes who engaged in regular physical activity, but not at an elite or competitive level. Individuals taking regular medication, except for oral contraceptives, and female subjects in the menstrual phase were excluded from the study. An activity index (Jansson & Hedberg, 1991) with values ranging between 5.5 and 20.5 was calculated to estimate the physical activity during leisure time and was 16 ± 3. The percentage of body fat, fat‐free body mass, and fat mass were estimated from skinfold measurements (triceps, biceps, and subscapular; (Durnin & Womersley, 1974)) and were 24 ± 8%, 52 ± 11 kg, and 16 ± 5 kg, respectively.

### 2.2 Experimental protocol

**FIGURE 1:**
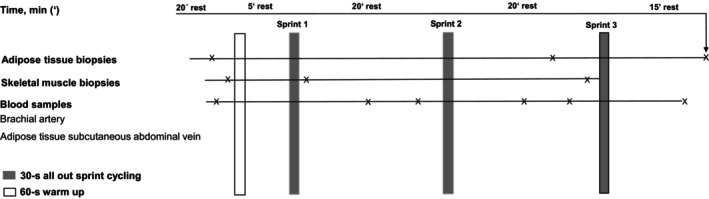
Schematic overview of the experimental protocol.

Subjects were asked to refrain from any strenuous exercise for 24 h prior to the experiment. Subjects reported to the laboratory between 08:00 am and 03:00 pm after an overnight fast. Subjects were instructed to consume 1–2 slices of white bread and water one to 2 h before the experiment. Subjects who reported after lunch were also instructed to consume a slice of bread and water in the morning. A Teflon catheter was inserted percutaneously into the brachial artery and a superficial subcutaneous adipose tissue vein on the anterior abdominal wall providing access venous drainage from the subcutaneous abdominal adipose tissue (Frayn et al., 1989; Simonsen et al., 1994; Summers et al., 1998). Five mL of blood was sampled from each of the artery and the adipose tissue vein, at rest before 60‐s warm up and 9 and 18 min after each sprint, with the subject in the supine position (Figure 1).

Subcutaneous abdominal adipose tissue biopsies were performed under local anesthesia without adrenaline by using a Hepafix needle (Braun Medical, Melsungen, Germany): before sprint 1, 15 min after sprint 2 and sprint 3. The first and last biopsies were performed on different sides of the umbilicus. Percutaneous muscle biopsies from quadriceps femoris muscle (vastus lateralis) were performed under local anesthesia, before sprint 1, immediately after sprint 1 and 18 min after sprint 2 (Bergström, 1975). Samples were frozen directly in liquid nitrogen and stored at −70 degrees C for later analysis. Data on abdominal venous ammonia, interleukine‐6 and glycerol (Esbjörnsson et al., 2006, 2023), skeletal muscle amino acids, ammonia, adenosine triphosphate (ATP), inosine monophosphate (IMP) and lactate as well as plasma amino acids have been presented elsewhere (Esbjörnsson, Rooyackers, et al., 2012).

### 2.3 Sprint exercise protocol

After a 60‐s warm‐up at a load of 25% of the individual braking load, the subjects performed three 30‐s “all‐out” cycle sprints (Bar‐Or, 1987) on a mechanically braked cycle ergometer (Cardionics, Sweden) with 20 min rest between sprints and were instructed to pedal as fast as possible at a load of 0.075 kilopond × kg body mass^−1^. Peak power (i.e., the highest 5‐s power) and mean power (the average power during the 30‐s cycle) were calculated for each of the three 30‐s cycle sprints. This experimental protocol, with relatively long rest periods between bouts, which was originally designed to achieve almost complete recovery of power output between sprints (Esbjörnsson et al., 1993), is a well‐characterized protocol for the metabolic and hormonal response to sprint exercise in both sexes (Esbjörnsson et al., 2006, 2009; Esbjörnsson, Rooyackers, et al., 2012; Esbjörnsson, Rundqvist, et al., 2012; Esbjörnsson‐Liljedahl et al., 2002; Rundqvist et al., 2017, 2019).

### 2.4 Adipose tissue biopsy preparation and analysis

Subcutaneous adipose tissue samples obtained before and after exercise were immediately blotted, placed on a plastic filter (Sefar Nitex 06‐210/233 Bigman AB, Sweden), rinsed in sterile saline, cut into smaller pieces and frozen in liquid nitrogen. Samples were stored at −80°C until processed. Due to limited biopsy sample material, analyses of purines and gene expression were performed on separate individuals in some cases, resulting in only three individuals having both RNA and purine analysis. However, an overlap of seven individuals was present for plasma and skeletal muscle purines and RNA. Frozen adipose tissue samples obtained at rest and 15 min after sprint 2 and sprint 3 were homogenized using a mini bead beater (Biospec Products, Bartlesville, Oklahoma, USA) and deproteinized in 6.5% sulfosalicylic acid and purines were analyzed by HPLC (Norman et al., 1991) in nine subjects (five females and three males). RNA was isolated from 10 to 20 mg of frozen tissue samples obtained at rest and 15 min after sprint 3 from nine subjects (six females and three males). Samples were treated with a homogenizer dispenser (Polytron, Kinematica AG, Lucerne, Switzerland) and a standard TRIzol® protocol (InvitrogenTM Life Technologies, Carlsbad, CA, USA). Additional purification was performed using the RNeasy Mini Kit (QIAGEN, Redwood City, CA, US, Cat. No.74104). RNA was quantified using Nanodrop (Spectrometer ND‐1000: NanoDrop®, Thermo Scientific, Wilmington, DE, USA). RNA quality was assessed using an Agilent 2100 Bioanalyzer (Agilent Technologies, Santa Clara, CA, US). RNA integrity (RIN) values showed consistent and acceptable RNA quality among the samples, except for one sample with RIN 3.2 which was excluded from the results. However, the main results of the study were the same with or without this sample. Due to the generally low RNA content of the samples, the Ovation® Pico WTA System (NuGEN Technologies Inc. San Carlo, CA, USA) was used to prepare amplified cDNA targets from 25 ng total RNA. The WT‐Ovation Exon Module (NuGEN Technologies Inc. San Carlo, CA, USA) was used to generate sense transcript cDNA, suitable for fragmentation and labeling. Fragmented and labeled single‐stranded cDNA for hybridization to the Human Gene 1.0 ST Array (Thermo Fisher Scientific Inc., Waltman, MA, USA, Cat. No. 901086) was generated using the Encore Biotin Module (NuGEN Technologies Inc. San Carlo, CA, USA). All three modules were used according to the manufacturer's instructions. Arrays were hybridized for 16 h at 45°C in a Gene Chip Oven 640 (Affymetrix, Santa Clara, CA, USA) followed by washing and staining using the Gene Chip Fluidics Station 450. Scanning was performed using the Affymetrix Gene Chip Scanner 3000 7G. Raw intensity data (CEL files) were processed by using Affymetrix Expression Console Software (v.1.0). Background correction (PM‐6CBG), global median normalization, and summarization of probe intensity (Plier) were performed. Global gene expression was first analyzed using Principal Components Analysis (PCA) where separation of pre versus post samples were assessed using paired *t*‐tests.

Differentially expressed genes in response to exercise were identified using the paired *t*‐test. Cutoffs were applied at fold change (FC) +/− 1.2 and false discovery rate (FDR) 5%. QIAGEN's Ingenuity® Pathway Analysis (IPA®, QIAGEN Redwood City, CA, USA) was used to identify pathways that were significantly enriched in the list of differentially expressed genes. The Human Gene 1.0 ST array was selected as the reference. Pathway analysis was conducted by Gene Set Enrichment Analysis (GSEA) based on Wikipathways 2024 (Agrawal et al., 2024) ontology data using the ClusterProfiler library (Xu et al., 2024). In order to test for pre vs. post exercise differences in the amount of blood obtained in the tissue samples gene expression of markers of blood contamination (*AHSP, ALAS2, GYPB, HBB, HBD, HEMGN, RHD, SLC4A1*) (Joehanes et al., 2012) was specifically analyzed on the microarray and none were found to be altered by the exercise. All raw data (CEL files) have been deposited in NCBI's Gene Expression Omnibus (Edgar et al., 2002) and are accessible via GEO Series accession number GSE267959.

### 2.5 Skeletal muscle biopsy preparation and analysis

Frozen muscle tissue was homogenized using a minibead beater (Biospec Products, Bartlesville, Oklahoma, USA) and deproteinized in 4% sulfosalicylic acid. Adenine nucleotide breakdown products were analyzed by HPLC in 15 subjects (Norman et al., 1991).

### 2.6 Blood/plasma preparation and analyses

After collection, blood samples from all subjects were centrifuged at 4°C to separate plasma. The plasma supernatant was frozen and stored at −70°C until analyzed for glucose by an enzymatic glucose oxidase method using a Beckman Coulter Oxygen Electrode (LX‐20, Brea, CA, USA), free fatty acids (FFA) by an enzymatic colorimetric assay (FUJIFILM Wako Chemicals Europe GmbH, Neuss, Germany Cat No 34‐91795, 436‐91995, and 270‐77000) and the purines inosine, hypoxanthine, and uric acid in a neutralized perchloric acid extract by HPLC (Norman et al., 1991). Xanthine levels were not reported because of technical problems in the HPLC analysis. However, the levels and temporal changes of plasma inosine, hypoxanthine, and uric acid were very similar to those obtained in a previous study using the same experimental protocol and HPLC method, supporting the accuracy of the measurements of inosine, hypoxanthine, and uric acid in the present study (Esbjörnsson‐Liljedahl et al., 2002). Lactate was determined in neutralized perchloric acid extract of whole blood using a fluorometric enzymatic method (Lowry & Passonneau, 1972).

### 2.7 Statistics

Values in the text are mean and SD unless otherwise stated. *p*‐values were accepted as statistically significant at *p* < 0.05. Gene expression data were analyzed by IPA (see above and results). The number of subjects was too small (*n* = 9) to look for sex‐related differences of exercise‐induced response in gene expression. Therefore, all data, inclusive plasma and power output variables (*n* = 15), were presented without dividing by sex and a one‐way repeated‐measures ANOVA (time) was used to test the effect of time for plasma and power output variables. However, sex differences regarding changes in plasma variables by time were shortly summarized in results as based on a two‐way repeated‐measures ANOVA (sex and time). Student's *t*‐test for paired observations or groups was used when appropriate. Statistical analysis of the relationship between metabolites in various tissues was performed using a Pearson's single linear regression analysis.

## 3 RESULTS

Peak power and mean power were 723 ± 170 and 562 ± 121 Watt, respectively, during the first sprint and decreased by 7% and 4%, respectively, over the three sprints (*p <* 0.01 and *p <* 0.01).

### 3.1 Metabolic measurements

**FIGURE 2:**
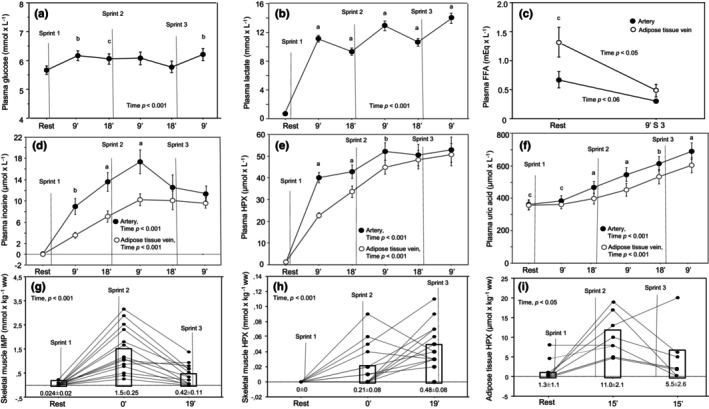
(a) Plasma glucose (*n* = 15), (b) plasma lactate (*n* = 15), (c) plasma FFA (*n* = 4), (d) plasma inosine (*n* = 13), (e) plasma hypoxanthine (HPX, *n* = 14), (f) plasma uric acid (*n* = 14), (g) skeletal muscle inosine monophosphate (IMP, *n* = 14), (h) skeletal muscle HPX (*n* = 13) and (i) adipose tissue HPX (*n* = 7) during repeated bouts of sprint exercise. “Time” denotes the main effect of time over all points of time tested by repeated‐measures ANOVA, n number of subjects and ww denotes wet weight. Values are mean ± SE. In figure A‐B the statistical level of differences between rest and the different time points were indicated by a = *p* < 0.001, b = *p* < 0.01, or c = *p* < 0.05. In figure c–f differences between arterial and adipose tissue venous concentrations were indicated by a = *p* < 0.001, b = *p* < 0.01, or c = *p* < 0.05.

Arterial plasma glucose, lactate, inosine, hypoxanthine, and uric acid increased during the sprint exercise session (Figure 2a,b,d–f). The levels of inosine, hypoxanthine, and uric acid were also measured in the vein draining adipose tissue and were generally lower than in the artery during the exercise session (Figure 2d–f). Arterial and adipose tissue venous FFA were measured at rest and 9 min after sprint 3 in four subjects to confirm the results of our previous studies of a decrease in systemic FFA during the exercise session. A decrease from rest to 9 min after sprint 3 was found in all four subjects in both the arterial and adipose tissue venous blood. In addition, the calculated arterial‐adipose tissue venous FFA difference also decreased from rest to 9 min post sprint 3 in all subjects (Figure 2c).

Skeletal muscle IMP (time *p* < 0.001), inosine (time *p* < 0.001), and hypoxanthine (time *p* < 0.001) increased during the sprint exercise session (Figure 2g,h). Values for inosine are not given in Figure 2 and were 0.003 ± 0.01, 0.015 ± 0.02, and 0.085 ± 0.06 mmol × kg ^−1^ ww at rest, immediately after and 9 min after sprint 2. No xanthine or uric acid was detected in skeletal muscle.

**FIGURE 3:**
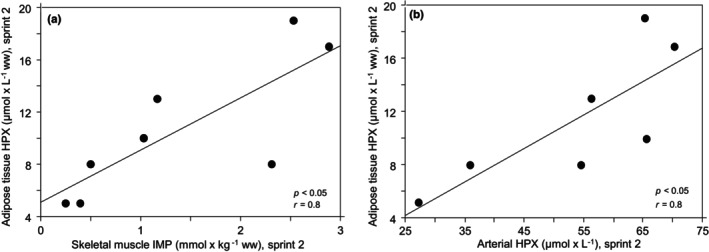
Relationship between (a) adipose tissue hypoxanthine (HPX) 15 min after sprint 2 and skeletal muscle inosine monophosphate (IMP) directly after sprint 2 and between (b) adipose tissue HPX 15 min after sprint 2 and plasma HPX 9 min after sprint 2 during repeated bouts of sprint exercise; ww denotes wet weight.

Adipose tissue hypoxanthine (time *p* < 0.05) also increased during the sprint exercise session (Figure 2i). The effect of time on adipose tissue xanthine was not significant, mainly due to a large SD for postexercise xanthine. When comparing resting and average xanthine 15 min after sprint 2 and 15 min after sprint 3, xanthine in adipose tissue increased from 1.6 ± 1.2 to 3.7 ± 2.4 μmol × kg^−1^ wet weight, (*p* < 0.05). No uric acid was found in adipose tissue. Positive correlations were found between adipose tissue hypoxanthine at 15 min after sprint 2 on the one hand and skeletal muscle IMP immediately after sprint 2 (*p* < 0.05), (Figure 3a) and arterial plasma hypoxanthine 9 min after sprint 2 on the other (*p* < 0.05), (Figure 3b).

### 3.2 Gene expression was downregulated by sprint exercise

**FIGURE 4:**
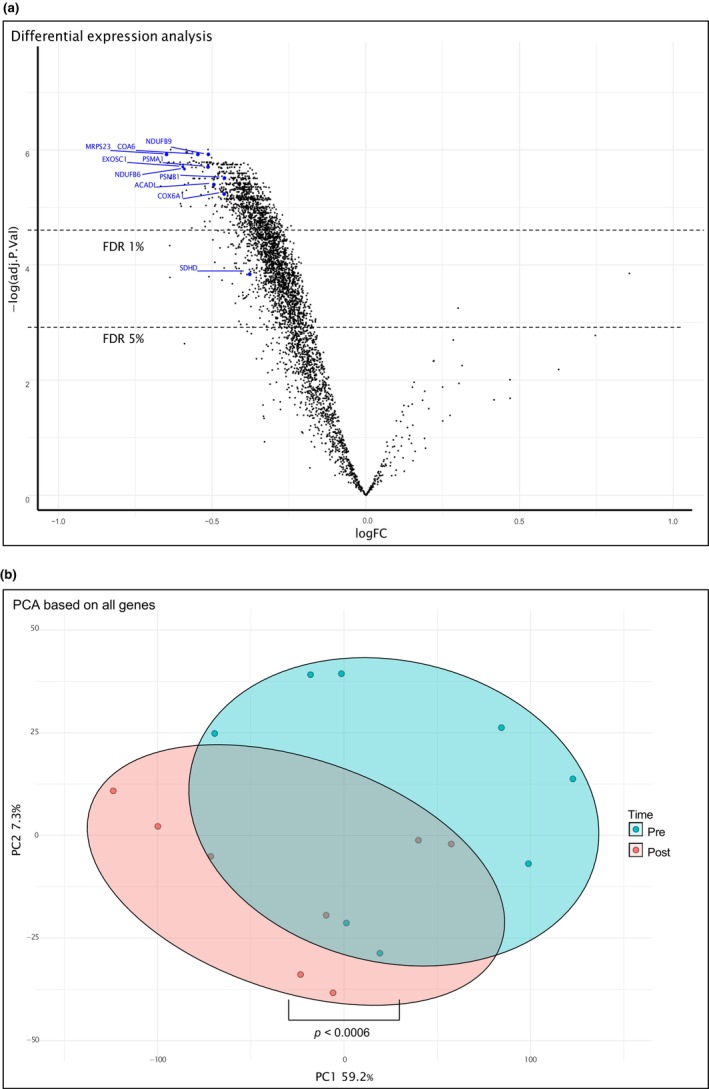
(a) Vulcano‐plot showing pre‐ versus post‐exercise log2 fold changes (x‐axis) along with ‐log false‐discovery rate from the differential expression analysis. 952 genes were differentially expressed at a false‐discovery rate of <5%. Microarray data from adipose tissue before and after sprint exercise in eight subjects. (b) The impact of exercise on global gene‐expression was assessed though Principal Component Analysis (PCA) based on all transcripts. There was a significant difference in the loading of principal component 1 between pre‐ and post‐exercise samples (*p* < 0.001). The figure denotes loading of individual samples on principial component 1 (x‐axis) and 2 (y‐axis) with 95% confidence ellipses.

Genes that were differentially expressed in adipose tissue 15 min after the last sprint were identified by gene expression array analysis. With a cutoff fold change (FC) of +/−1.2 and a false discovery rate (FDR) of less than 5%, 952 differentially expressed genes were identified by IPA, of which two were upregulated and 950 were downregulated (Figure 4a). A PCA plot revealed a shift in gene expression by sprint exercise (Figure 4b). Many of the downregulated genes (FC −1.2 to −1.6) were related to the function of mitochondria (e.g., *ATP synthases*, *NADH:ubiquionone oxidoreductase*, *cytochrome c oxidases*, *SDHB*, and *SDHC*), proteasomes (e.g., *PSMA1*, *PSMA3*, *PSMA6*, and *PSMA7*) and ribosomes (various *MRPS* and *MRPL*). The 952 genes are listed in Table S1. See also the Vulcano plot (Figure 4a). Very few upregulated genes were identified at the FDR of 5%. Using a more generous FDR of 15%, 39 genes were found to be upregulated (FC 1.2–2.4) and are presented in Table S2. Some of these genes were related to oxidative stress and inflammation (e.g., *IER3* and *TGFB1*).

### 3.3 Pathway analysis

**FIGURE 5:**
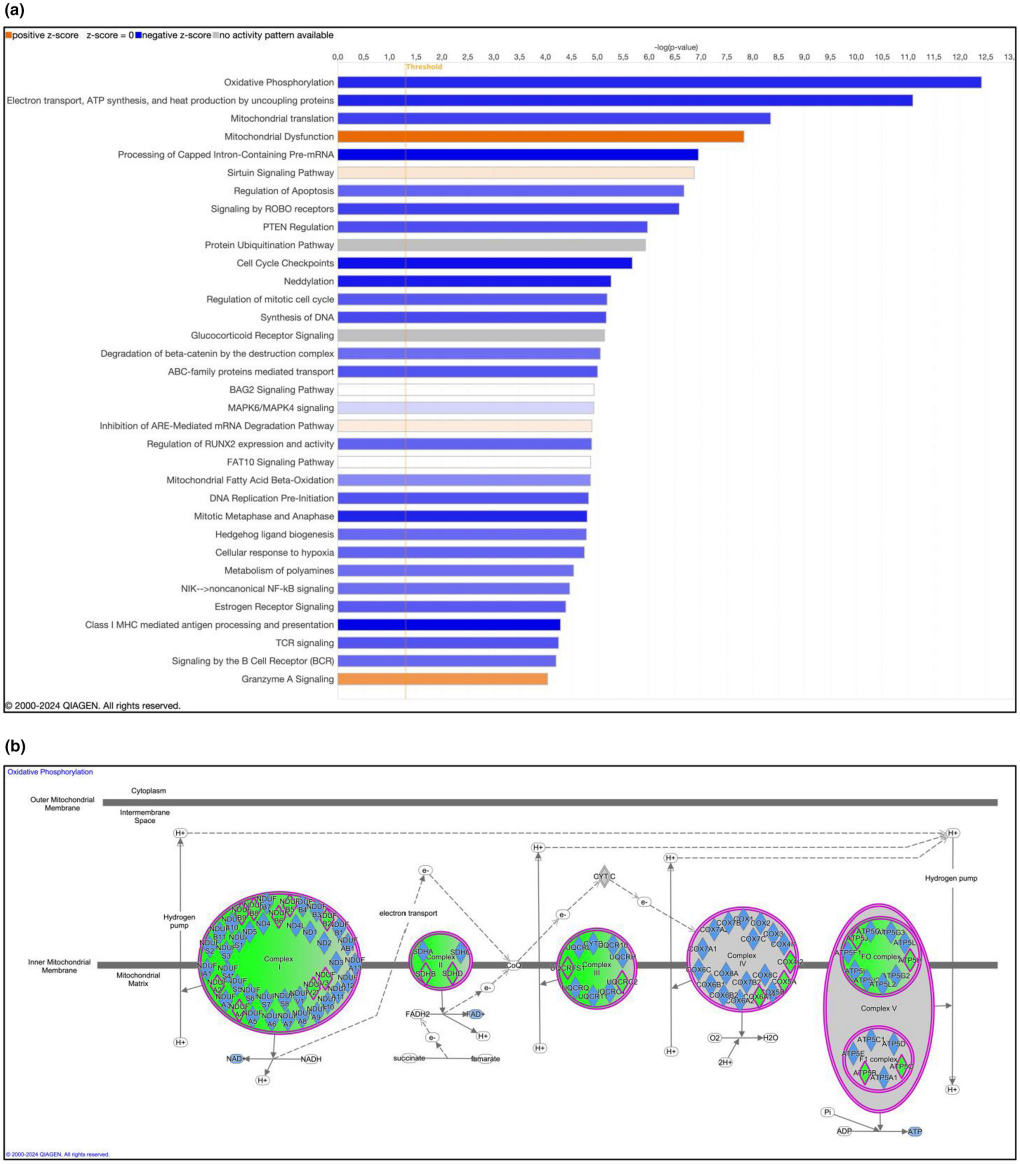
(a) Pathways as identified by the IPA from 952 differentially expressed genes in adipose tissue after sprint exercise in eight subjects, z‐score >2 or <−2 and ‐log(*p* value) >4. (b) Illustration of the predicted inhibition of the electron transport chain with affected enzymes and proteins in purple and green diamonds as suggested from the differentially expressed genes The full names of these genes are found in Table 1.

In response to the acute sprint exercise, significantly decreased signaling (z‐score >2 and −log(*p*‐value) >4) was predicted by the IPA toolkit for mitochondrial function related pathways such as oxidative phosphorylation, electron transport, ATP synthesis, and heat production by uncoupling, mitochondrial translation, and mitochondrial fatty acid beta oxidation. In addition, several other pathways were predicted to be inhibited such as RNA‐ and DNA‐ handling, cell‐cycle regulation, and apoptosis. An increased signaling was predicted for mitochondrial dysfunction and granzyme A signaling. Some pathways were also predicted to be significantly regulated, but the direction could not be clearly determined (z‐score between −2 and 2) such as sirtuin signaling, protein ubiquitination, AU‐rich elements (ARE)‐mediated mRNA decay, and glucocorticoid receptor signaling (Figure 5a,b) and Table S3). The predicted inhibition of oxidative phosphorylation was based on 27 differentially expressed genes, representing all five complexes in the electron transport chain (Figure 5b and Table 1). All genes behind the predicted inhibited or activated pathways are given in Table S3.

**TABLE 1:** Differentially expressed genes predicting an inhibition of oxidative phosphorylation in adipose tissue after sprint exercise in eight subjects analyzed by IPA, z‐score = −5.20 and −log(*p* value)= 12.4.

Symbol	Entrez gene name	Affymetrix	*p* Value	Fold change	False discovery rate
ATP5F1B	ATP synthase F1 subunit beta	7964234	4.29 × 10^−4^	−1286	3.82 × 10^−2^
ATP5F1C	ATP synthase F1 subunit gamma	7926084	1.55 × 10^−4^	−1320	4.57 × 10^−2^
ATP5PD	ATP synthase peripheral stalk subunit d	8018288	1.65 × 10^−3^	−1247	4.72 × 10^−2^
ATP5PF	ATP synthase peripheral stalk subunit F6	8069633	7.88 × 10^−4^	−1276	4.29 × 10^−2^
ATP5PO	ATP synthase peripheral stalk subunit OSCP	8070160	2.20 × 10^−3^	−1290	4.97 × 10^−2^
COX4I2	Cytochrome c oxidase subunit 4I2	8061572	1.87 × 10^−3^	−1313	4.77 × 10^−2^
COX5A	Cytochrome c oxidase subunit 5A	7990436	1.24 × 10^−3^	−1214	4.53 × 10^−2^
COX6A1	Cytochrome c oxidase subunit 6A1	8119153	5.23 × 10^−4^	−1360	3.92 × 10^−2^
CYB5A	Cytochrome b5 type A	8023855	3.12 × 10^−4^	−1259	3.98 × 10^−2^
NDUFA2	NADH:ubiquinone oxidoreductase subunit A2	8114618	1.05 × 10^−5^	−1370	3.32 × 10^−2^
NDUFA4	NDUFA4 mitochondrial complex associated	8138224	1.62 × 10^−3^	−1284	4.72 × 10^−2^
NDUFAB1	NADH:ubiquinone oxidoreductase subunit AB1	8000323	4.61 × 10^−4^	−1375	3.82 × 10^−2^
NDUFB2	NADH:ubiquinone oxidoreductase subunit B2	813602	1.12 × 10^−3^	−1261	4.55 × 10^−2^
NDUFB4	NADH:ubiquinone oxidoreductase subunit B4	8093314	9.32 × 10^−4^	−1317	4.31 × 10^−2^
NDUFB5	NADH:ubiquinone oxidoreductase subunit B5	8084092	8.44 × 10^−4^	−1343	4.27 × 10^−2^
NDUFB6	NADH:ubiquinone oxidoreductase subunit B6	8160587	5.21 × 10^−4^	−1466	3.92 × 10^−2^
NDUFB8	NADH:ubiquinone oxidoreductase subunit B8	7935810	2.10 × 10^−3^	−1263	4.91 × 10^−2^
NDUFB9	NADH:ubiquinone oxidoreductase subunit B9	8148270	1.78 × 10^−4^	−1411	4.24×10^−2^
NDUFS6	NADH:ubiquinone oxidoreductase subunit S6	8104298	1.82 × 10^−3^	−1320	4.76 × 10^−2^
NDUFV2	NADH:ubiquinone oxidoreductase core subunit V2	8039068	3.90 × 10^−4^	−1306	3.86 × 10^−2^
NDUFV3	NADH:ubiquinone oxidoreductase subunit V3	8068857	5.88 × 10^−4^	−1248	4.12 × 10^−2^
SDHB	Succinate dehydrogenase complex iron sulfur subunit B	7912928	1.33 × 10^−3^	−1281	4.58 × 10^−2^
SDHD	Succinate dehydrogenase complex subunit D	7943853	1.44 × 10^−3^	−1340	4.67 × 10^−2^
SURF1	SURF1 cytochrome c oxidase assembly factor	8164896	1.24 × 10^−3^	−1359	4.53 × 10^−2^
UQCRC2	Ubiquinol‐cytochrome c reductase core protein 2	7993872	1.89 × 10^−4^	−1251	4.18 × 10^−2^
UQCRFS1	Ubiquinol‐cytochrome c reductase, Rieske iron–sulfur polypeptide 1	8035880	1.67 × 10^−3^	−1412	4.75 × 10^−2^
UQCRH	Ubiquinol‐cytochrome c reductase hinge protein	7901212	5.40 × 10^−4^	−1396	3.98 × 10^−2^

*Note*: © 2000–2024 QIAGEN. All rights reserved.

**FIGURE 6:**
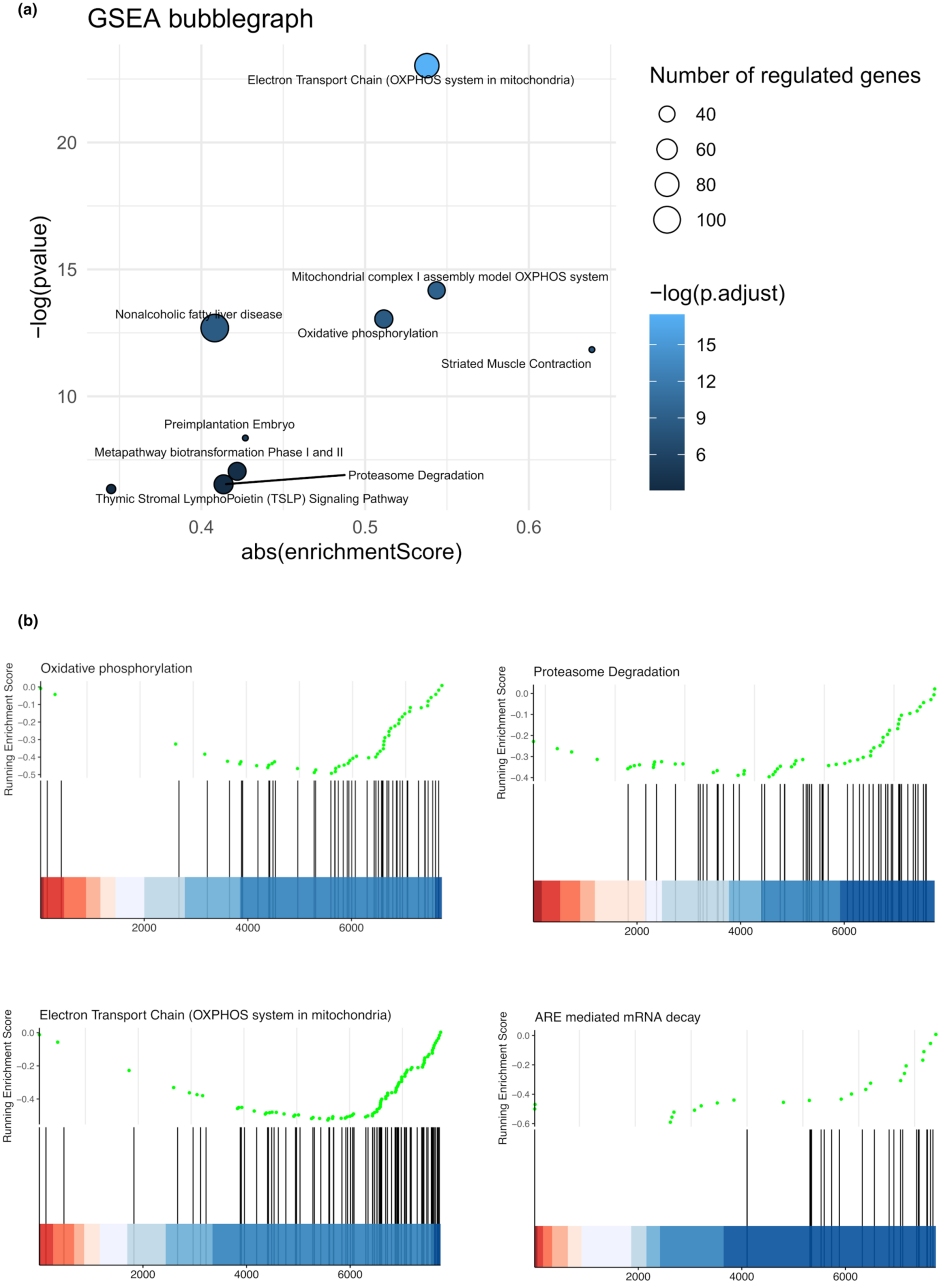
(a) Bubble‐graph of the Gene Set Enrichment Analysis (GSEA) showing enrichment score (x‐axis) along with ‐log false‐discovery rate (y‐axis) of significantly enriched pathways. The size of the bubbles denotes the number of genes belonging to each pathway. (b) Barcode graphs showing the distribution of the exercise induced fold‐changes and enrichment scores of the genes belonging to each pathway in relation to all analyzed genes. All significant enriched pathways had a strong negative enrichment score indicative of a robust transcriptional downregulation after exercise.

GSEA revealed a significant downregulation of nine pathways (FDR <5%) with Electron Transport Chain (OXPHOS system in mitochondria) being the most robustly downregulated pathway (Figure 6a,b and Table 2).

**TABLE 2:** Pathways as identified by Gene Set Enrichment Analysis from 952 differentially expressed genes in adipose tissue after sprint exercise in eight subjects, FDR <5%.

Description	Set size	Enrichment score	*p* Value	*p* Adjust
Electron transport chain (OXPHOS system in mitochondria)	83	−0.54	1 × 10^−10^	3 × 10^−8^
Mitochondrial complex I assembly model OXPHOS system	45	−0.54	7 × 10^−7^	9 × 10^−5^
Oxidative phosphorylation	49	−0.51	2 × 10^−6^	2 × 10^−4^
Nonalcoholic fatty liver disease	110	−0.41	3 × 10^−6^	2 × 10^−4^
Striated muscle contraction	22	−0.64	7 × 10^−6^	4 × 10^−4^
ARE‐mediated mRNA decay	22	−0.63	1 × 10^−5^	6 × 10^−4^
Preimplantation embryo	22	0.43	2 × 10^−4^	9 × 10^−3^
Metapathway biotransformation Phase I and II	48	−0.42	9 × 10^−4^	3 × 10^−2^
Proteasome degradation	53	−0.41	1 × 10^−3^	4 × 10^−2^
Thymic stromal lymphopoietin (TSLP) signaling pathway	24	0.34	2 × 10^−3^	5 × 10^−2^

The IPA pathway analysis predicted a significant regulation of ARE‐mediated mRNA decay, albeit with a low z‐score wherefore the directionality was uncertain. Therefore, the 22 genes belonging to this pathway (Table 3) was further analyzed as a gene‐set using GSEA. This indicated that a robust down‐regulation of this pathway (enrichment‐score −0.63 *p* < 0.001) and the resulting Barcode‐graphs is presented in Figure 6.

**TABLE 3:** Differentially expressed genes predicting a regulation of ARE‐mediated mRNA decay in adipose tissue after sprint exercise in eight subjects analyzed by IPA, z‐score = 0.63 and −log(*p* value) = 4.9.

Symbol	Entrez gene name	Affymetrix	*p* Value	Fold change	False discovery rate
AGO4	Argonaute RISC component 4	7900009	4.16 × 10^−4^	−1204	3.78 × 10^−2^
AKT3	AKT serinethreonine kinase 3	7925531	1.58 × 10^−4^	−1367	4.45 × 10^−2^
CNOT2	CCR4‐NOT transcription complex subunit 2	7957106	1.78 × 10^−3^	−1238	4.74 × 10^−2^
CNOT6	CCR4‐NOT transcription complex subunit 6	8110589	1.89 × 10^−3^	−1201	4.78 × 10^−2^
EXOSC1	Exosome component 1	7935462	1.54 × 10^−3^	−1468	4.69 × 10^−2^
PAPOLA	Poly(A) polymerase alpha	7976598	8.70 × 10^−4^	−1253	4.23 × 10^−2^
PPP2CB	Protein phosphatase 2 catalytic subunit beta	8150126	8.15 × 10^−4^	−1279	4.22 × 10^−2^
PPP2R5A	Protein phosphatase 2 regulatory subunit B'alpha	7909586	1.18 × 10^−3^	−1253	4.55 × 10^−2^
PRKAG1	Protein kinase AMP‐activated non‐catalytic subunit gamma 1	7962935	1.07 × 10^−4^	−1263	4.48 × 10^−2^
PSMA1	Proteasome 20S subunit alpha 1	7946728	6.56 × 10^−4^	−1409	4.12 × 10^−2^
PSMA3	Proteasome 20S subunit alpha 3	7974603	1.99 × 10^−3^	−1357	4.82 × 10^−2^
PSMA6	Proteasome 20S subunit alpha 6	7973936	1.89 × 10^−3^	−1307	4.77 × 10^−2^
PSMA7	Proteasome 20S subunit alpha 7	8067382	1.44 × 10^−3^	−1322	4.65 × 10^−2^
PSMB1	Proteasome 20S subunit beta 1	8130952	1.04 × 10^−3^	−1355	4.47 × 10^−2^
PSMB3	Proteasome 20S subunit beta 3	8006812	1.88 × 10^−3^	−1401	4.79 × 10^−2^
PSMB7	Proteasome 20S subunit beta 7	8164067	6.26 × 10^−4^	−1323	4.08 × 10^−2^
PSMC1	Proteasome 26S subunit, ATPase 1	7976189	1.34 × 10^−3^	−1243	4.56 × 10^−2^
PSMC6	Proteasome 26S subunit, ATPase 6	7974380	2.10 × 10^−3^	−1239	4.90 × 10^−2^
PSMD6	Proteasome 26S subunit, non‐ATPase 6	8088535	1.11 × 10^−4^	−1311	4.33 × 10^−2^
PSMD7	Proteasome 26S subunit, non‐ATPase 7	7997230	2.70 × 10^−4^	−1293	3.93 × 10^−2^
PSMD14	Proteasome 26S subunit, non‐ATPase 14	8045946	1.63 × 10^−4^	−1242	4.36 × 10^−2^
TNFSF10	TNF superfamily member 10	8092169	8.77 × 10^−4^	−1449	4.23 × 10^−2^

*Note*: © 2000–2024 QIAGEN. All rights reserved.

### 3.4 Upstream analysis

Predicted increase in activity state was identified by the IPA toolkit for 23 factors (z‐score cut off at 2 and a *p* value cutoff at 0.01). The most common types of upstream activators were microRNA (mir‐1, mir‐194, mir‐802, mir‐9‐5p, mir‐144‐5p, mir‐124‐3p, and mir‐450a‐5p) followed by transcriptional regulators (ZHX2, BACH1, CLPB, SERTAD2, and KLF3). In addition, the ligand‐dependent nuclear receptor NR4A1, the peptidase CLPP, the translation regulator LARP1, the cytokine CLCF1, the enzymes CPT1B and CA9, and RICTOR. SAMMSON, CST5, MKKS, and HAX1, denoted as “others”, were predicted as upstream regulators (Table S4). A decrease in activity was predicted for 27 factors (z‐score cutoff at −2 and a *p* value cutoff at 0.01). The most common types of upstream inhibitors were transcriptional regulators (TEAD1, PPARGC1A, PPARGC1B, NRF1, NFE2L1, KLF15, LMX1A, LMX1B, H2AX, and XBP1) followed by transmembrane receptors (PLA2R1, IGF1R, CD247, and CD40), enzymes (DDX5, GFER, and YARS2), and kinases (TRIB1, STK11, and CAB39L). In addition, the ligand‐dependent nuclear receptor ESRRA, the phosphatase IGBP1, the transporter HBA1/HBA2 as well PERM1, WTAP, PTCD1, and UMOD, denoted as “others”, were predicted as upstream regulators (Table S4).

### 3.5 Sex differences

The number of subjects was too small to look for sex‐related differences in the exercise‐induced response in adipose tissue gene expression. Therefore, all data were presented without dividing by sex. However, the larger number of subjects for blood and skeletal muscle variables allowed a sex‐related statistical analysis. Interaction between time and sex or general effect of sex were found for arterial and adipose tissue venous inosine and xanthine and for IMP and hypoxanthine in skeletal muscle. The interactions were explained by a lower exercise‐induced increase in females and the general effects of sex by lower values in females. The IMP data have previously been reported (Esbjörnsson et al., 2006; Esbjörnsson, Rooyackers, et al., 2012), but not the correlations to adipose tissue hypoxanthine (Figure 3).

## 4 DISCUSSION

### 4.1 Key findings

To the best of our knowledge, this is the first study on the acute response of the adipose tissue transcriptome to SIT. The study did not confirm the hypothesis of an increased expression of genes specifically related to lipolysis, re‐esterification, thermogenesis, or mitochondria in the early phase after acute sprint exercise. Instead, a downregulation of several gene sets was shown with a focus on transcripts related to oxidative metabolism, consistent with the predicted downregulation of mitochondrial transcriptional coactivators such as PPARGC1A, PPARGC1B, and PERM1. In addition, a downregulation of proteasome, ribosome, and cell cycle‐related genes was also found, as well as the predicted downregulation of the proteasome transcriptional activator NFR1. Downregulation of genes related to mitochondria and proteasomes may be related (Maharjan et al., 2014) and both organelles may develop dysfunction due to oxidative stress (Crawford et al., 1997; Diaz‐Ruiz et al., 2015).

### 4.2 No support for increased thermogenesis or browning in adipose tissue after SIT

In the present study, no evidence of increased thermogenesis was observed as is often seen in rodents exposed to exercise (Stroh & Stanford, 2023). Thus, an unanswered question is why SIT leads to reduced body fat mass. Therefore, we need to further examine the temporal changes in gene expression in adipose tissue after exercise, as only the early postexercise phase was analyzed in the current study. In addition, we also need to explore post transcriptional activation of thermogenesis. Moreover, the search for the contribution of organs other than white adipose tissue to the sprint exercise‐induced thermogenesis, such as liver, skeletal muscle, and brown adipose tissue is also warranted. Exercise‐induced changes in gene expression in skeletal muscle were not analyzed in the present study but have been done by others. While transcripts and pathways associated with oxidative and fat metabolism seems to be acutely downregulated in adipose tissue as found in the present study and by others (Ludzki et al., 2021), they are upregulated in skeletal muscle (Dreher et al., 2023; Rundqvist et al., 2017). Moreover, a decrease or no change in mitochondrial respiratory capacity or enzyme activity in adipose tissue but an increase in skeletal muscle has been demonstrated after chronic high intensity interval training (Dohlmann et al., 2018; Larsen et al., 2015). Recently, also a discordant pattern in adipose tissue and skeletal muscle of gene expression was found after strength exercise combined with endurance training (Svensson et al., 2024). Upregulation in skeletal muscle of oxidative and fat metabolism may contribute to an increase in postexercise energy consumption (Borsheim & Bahr, 2003; Townsend et al., 2017). In brown adipose tissue, thermogenesis could be activated after SIT by increasing postexercise FFA level in plasma (Bülow, 2016; Rundqvist et al., 2017). Also, interorgan futile cycles may contribute to energy expenditure and metabolic regulation as recently reviewed (Sharma et al., 2024). Finally, a reduced energy intake due to decreased appetite (Islam et al., 2017) cannot be excluded and may contribute to an increased energy deficit and in its turn to a reduce body fat mass after chronic SIT.

### 4.3 Other studies with downregulation of AT gene expression

Two recent studies on the transcriptomic response to acute exercise found similar trends of downregulation of transcripts representing oxidative metabolism (Ludzki et al., 2021) or adipogenesis (Dreher et al., 2023; Ludzki et al., 2021), but the number of differentially expressed genes at similar cutoffs as in the present study was much lower in these two studies. However, these studies differed from our study in that they examined lower exercise intensities, overweight, or obese subjects and that the postexercise biopsy was performed 1 h after the last bout of exercise, whereas in the present study it was performed after 15 min. Furthermore, both studies (Dreher et al., 2023, Ludzki et al., 2021) showed an upregulation of gene sets involved in inflammation. In our study, only a few genes related to the inflammatory response were upregulated. Again, the shorter time between end of exercise and the postexercise biopsy in our study as compared to the earlier ones may help to explain that only a few inflammatory genes were upregulated in our study.

### 4.4 Potential explanations for the unexpected downregulation

An enrichment of downregulated pathways related to mitochondria was predicted from the differentially expressed genes. Mitochondria are known to produce free radicals, but are also sensitive to oxidative stress (Zhang et al., 1990). For example, Crawford et al. demonstrated in vitro that oxidative stress downregulated mitochondrial RNAs, but not cytoplasmic such as 18/28S rRNAs and concluded that this may represent a general mechanism to protect the cell from oxidative stress (Crawford et al., 1997). We would like to highlight FFA, IL‐6 and the purines hypoxanthine, xanthine and uric acid as potential sources of oxidative stress in adipose tissue during SIT and downregulation of mitochondria‐related pathways (Morel & Barouki, 1999; Raja et al., 2022; Saugstad, 1988; Sautin et al., 2007; Tumova et al., 2016). First, in a pilot study, the subcutaneous abdominal adipose tissue blood flow was measured with 133 Xe‐wash out and a marked decrease was found in all three subjects after the first 30‐s sprint (M. Esbjörnsson, J. Bülow, B. Norman and E. Jansson unpublished data; ethical approvement Dnr: 2015/2340‐32). Reduced adipose tissue blood flow may lead to entrapment of FFA in adipose tissue, as has been discussed previously (Hodgetts et al., 1991; Romijn et al., 1993) and recently especially in relation to enhanced lipolysis during SIT, as indicated by increased plasma glycerol in a subcutaneous adipose tissue vein (Esbjörnsson et al., 2023). In fact, entrapment was evident in earlier studies during SIT, where a decreased venous plasma FFA was found, followed by a postexercise increase in FFA (Rundqvist et al., 2017, 2019). These findings were extended in the current study, where a decrease of both arterial and adipose tissue venous FFA was observed during SIT. Second, adipose tissue venous IL‐6 increased markedly during SIT, reflecting increased IL‐6 in adipose tissue (Esbjörnsson et al., 2023). Finally, in the current study we have added analyses of purines in blood, adipose tissue, and skeletal muscle as markers of oxidative stress and found an increase in purines in all tissues during SIT. Of note, the final purine breakdown product uric acid is a potent pro‐oxidant in adipose tissue (Sautin et al., 2007) and uptake from blood into adipose tissue was indicated during SIT. However, the role of purines as a potential source of oxidative stress during SIT is unclear, as no uric acid was detected in adipose tissue in the current study, although increased levels of uric acid were found in both the arterial and adipose tissue venous plasma during SIT. In fact, in contrast to mice, a very low activity of xanthine oxidoreductase, the enzyme that converts xanthine to uric acid, was detected in human adipose tissue (Nagao et al., 2018).

Possible mechanisms, such as regulation at the transcriptional level or by a decay of mRNA (Hollams et al., 2002; Schoenberg & Maquat, 2012) were not specifically investigated in the present study. Yet, a downregulation of ARE‐mediated mRNA decay was indicated. Interestingly, ARE‐mediated decay has been shown to regulate gene expression through changes in oxygen level by an upregulation during hypoxia and a downregulation during reoxygenation (De Toeuf et al., 2018).

### 4.5 Short‐lasting downregulation?

The downregulation of genes related to mitochondria and fat metabolism seen after exercise has been suggested to be transient (Ludzki et al., 2021). Indeed, own preliminary data suggest that there was no pronounced downregulation of oxidative metabolism genes 2 h after SIT (M. Esbjörnsson, J. Bülow, B. Norman and E. Jansson unpublished data; ethical approvement Dnr: 2015/2340‐32). However, the changes in mitochondrial content, enzymes, and function after chronic high‐intensity interval exercise are inconsistent (Ahn et al., 2022; Dohlmann et al., 2018; Larsen et al., 2015), with indications of a decrease (Dohlmann et al., 2018; Larsen et al., 2015) no change (Larsen et al., 2015), or an increase (Ahn et al., 2022).

### 4.6 Methodological aspects

Differences in experimental protocols such as intensity and type of exercise, different populations such as obese or nonobese individuals, timing of the postexercise biopsy in relation to end of exercise, number of biopsies, and diurnal rhythms must be taken into account when comparing various studies of adipose tissue response to exercise (Dreher et al., 2023; Fabre et al., 2018; Guerrier et al., 2023; Loboda et al., 2009; Ludzki et al., 2021). The strategy in the present study was to perform the experiments at different times of the day to reduce the risk of a potential confounding effect due to a diurnal rhythm‐driven change in metabolic genes and also to avoid a potential repeated biopsy effect on gene expression. However, while it is a strength to avoid the effect of repeated biopsies, it limits studies of temporal changes in gene expression. Due to the lack of data on temporal changes in gene expression response in the literature, we explored the early response to acute exercise in this first study. Finally, we chose to study a young, healthy, nonobese population, that is, a similar population in which sprint exercise‐induced EPOC has been demonstrated (Hazell et al., 2012).

During SIT, relationships were found between the increase in skeletal muscle IMP, systemic hypoxanthine, and in adipose tissue hypoxanthine. This suggests that a significant portion of the variation in adipose tissue hypoxanthine, a marker of metabolic stress, is related to muscle metabolic stress and is less influenced by of other possible stress factors such as anesthesia, preceding biopsy, or venous catheterization. These relationships between metabolic changes in adipose tissue and skeletal muscle may indicate that skeletal muscle drives the changes in adipose tissue and thereby also the transcriptomic changes. However, the study is unpowered to test such an hypothesis.

It is not known in which cell types the downregulation of mitochondrial‐related genes occur in the adipose tissue. Most of the adipose tissue volume is occupied by adipocytes, even though many other cell types related to vasculature and immune function resides within adipose tissue. Single cell type analysis is needed to elucidate this question. However, mitochondria in adipocytes are known to have several important functions such as produce ATP for the FFA‐TG cycle, but also other metabolic cycles and metabolic pathways requiring ATP (Cai et al., 2023).

### 4.7 Strength and limitations

A notable strength is that we use a well‐characterized experimental protocol of the metabolic and hormonal response to sprint exercise in both sexes (Esbjörnsson et al., 2006, 2009; Esbjörnsson, Rooyackers, et al., 2012; Esbjörnsson, Rundqvist, et al., 2012; Esbjörnsson‐Liljedahl et al., 2002; Rundqvist et al., 2017, 2019). A limitation is that no non‐exercise control group was included in this pilot study. The finding of a downregulated gene expression related to oxidative metabolism needs to be confirmed in future studies, although recent evidence supports our findings (Ludzki et al., 2021). Also, the role of exercise intensity needs to be further explored by directly comparing, for example, various intensities. Furthermore, studies in obese subjects (Dahlman et al., 2006; Heinonen et al., 2017) have found a downregulation of similar gene sets as in our exercise study. This may indicate that these gene sets in adipose tissue are targets of common environmental stimuli such as oxidative stress in obesity or after acute exercise (Crawford et al., 1997; Manna & Jain, 2015; Morel & Barouki, 1999).

## 5 CONCLUSION

A downregulation of genes related to mitochondrial function, fat metabolism, proteasomes, ribosomes, and the cell cycle was found in subcutaneous adipose tissue in the early phase after repeated bouts of sprint exercise. Thus, contrary to the hypothesis, no signs of an increased thermogenesis were found. Whether this downregulation is a dysfunctional response or a potentially protective mechanism against SIT‐induced oxidative stress by temporarily inhibition of the mitochondria is not known.

## AUTHOR CONTRIBUTIONS

ME, EJ, and JB conceived and designed the research. ME, EJ, BN, HR, and JB performed experiments. BN and TÖ performed biochemical and microarray analyses. ME and EJ analyzed data. ER performed bioinformatic analyses. EJ, ME, BN, and JB interpreted results of experiments. ME and ER prepared figures and HR submitted raw data to GEO. EJ and ME drafted manuscript. EJ, ME, BN, HR, ER, and JB edited and revised the manuscript. All authors approved the final version of manuscript.

## FUNDING INFORMATION

This study was supported by grants from the Swedish Research Council for Sport Science (CIF), the Swedish Society of Medicine, Centre of Gender Related Medicine and the foundations of Sigurd & Elsa Goljes Minne (LA2022‐0092), O. E. & Edla Johansson, Åke Wiberg and Magnus Bergvall.

## CONFLICT OF INTEREST STATEMENT

The authors declare no conflict of interest.

## ETHICS STATEMENT

Subjects were fully informed about the procedures and potential risks of the experiment before giving written informed consent to participate in the study, which was approved by the Ethics Committee at Karolinska Institutet (Dnr 235‐00).

## Supporting information


Table S1.

Table S2.

Table S3.

Table S4.

## Data Availability

Transcriptomic data are available via the GEO database at NCBI (GSE267959). Additional study data are available upon request from the corresponding author.
